# Editorial

**Published:** 1903-02-15

**Authors:** 


### EDITORIAL.

THE Regents of New York State have declined to issue the Doctor of Dental Surgery degree to such as have the Master of Dental Surgery degree, without examination. This may be a disappointment to those who desired an exchange of degrees, but we believe it a prudent decision. The public good should always be paramount, and while we have no idea that a public injury would result directly had this proposition carried, it would have sou nsettled standards that have been erected at so much cost that it might possibly have been a harzardous experiment. As it now stands the way is open for such as may earnestly covet the degree of Doctor of Dental Surgery, to secure it by establishing his unquestioned right to it because of superior technical and scientific attainments.

AT the recent meeting of the Ohio State Dental Society the office of president was given to Dr. J. B. Beauman, of Columbus. This is an honor worthily bestowed, as Dr. Beauman has faithfully and conscientiously given many years and much valuable time to the practice of our profession in a dignified and professional way. Dr. Beauman began the practice of dentistry more than half a century ago, and it was refreshing to see the ardor he exhibiting in entering into the discussion of papers; also presenting a clinic on one of his favorite specialties. May he live longer to inspire us to nobler living and a more enthusiastic pursuit of our profession.

THE *Texas Dental Journal* and *Dental Clippings* are to be consolidated and issued under the auspices of the A. P. Carey Co., of Dallas. The publishers propose to enlarge the

Journal and issue it under the joint editorship of a chief and twelve associate editors living in various sections of the extreme south. It is proposed to publish all original matter of the territory and to discontinue reprinting any article. It is certainly a commendable thing to strive to gather together and put on permanent record the writings of the best workers in one's own territory, and we congratulate the publishers for undertaking this work. The suggestion might well be copied in other parts of the country. At the same time there are many good things which will bear reprinting in brief, at least, and the constituents of every Journal appreciate well selected reprints to which they might not have access in any other way. There is one feature connected with this consolidation which we can not so heartily commend, and this is the proposition to send the Journal without cost to every dentist in Texas and parts of adjoining states. The small fee prevailing for our most excellent Journals ought not prevent anyone who is worthy of professional recognition from having at least one Journal; and the probabilities are that a man who is so mean that he will not pay a dollar for a well conducted Dental Journal will not appreciate one that is laid upon his desk without cost.

THE reduction in subscription price is not the only change that has taken place in the dental periodical world, with the beginning of the new year, 1903 volumes. The *Dental Cosmos* has changed form completely, by increasing the size of its page and setting its type in double column, except the editorial pages. A new cover design completely obliterates all traces of the tasteful and dignified form in which this Journal has month after month presented itself for more than forty years. Typographically, the new form may be an improvement, and we hope to become accustomed to the new arrangement of matter in time. We are glad, however, that the name was left unchanged.

THE *International* sticks to its old price of two dollars and a half per; but it has turned *yellow*, not, however, from age nor from having abandoned its usual high ethical standards. It's only the cover, inside the matter is better than ever and the new paper and press-work is all that could be desired. May it live long and prosper.

---

THE *Dominion Journal* comes out in a handsome new green cover but the paper used in this Journal could be changed without causing a very serious injury to the eyesight of its readers. It is scarcely as good as that used in the *Digest*, which by the way, has improved by placing its table of contents on the front cover page; though we don't see why it should be printed in red ink. The December number carried nearly one hundred pages of advertising, perhaps this is the reason its publishers can't use better paper.

---

THE *Dental Summary's* January cover is the worst yet. But it makes up for it by a remarkable symposium on porcelain work. Extra pages are added to accomodate the original contributions from a large number of porcelain workers and a good collection of brief extracts from the current literature of the past year.

---

FOR real genuine simon pure art the *Items* leads all the rest with its cover printed in red and black and a quarter century embossed silver medal in one corner. We expected on turning its leaves to find the amalgam question again opened for discussion; but instead we face a pair of tin plated Knights with Chinese battle axes crossed over a strong looking portal marked "Exclusive Contributions." We are almost afraid to turn to the next pages, but a little Juniper tree down in one corner with a sign board across its trunk carries the cabalistic word JAN, which suggests that it is the pass word, we give it under breath, and turn the page to be confronted with a pair of those horrible puzzle pictures in which you are requested to find George Washington and Carrie Nation with their little hatchets. Unfortunately the printer neglected to put the nècessary instructions under the pictures, and after viewing them in every conceivable light and from every direction without a single impression our eye catches sight of our pass word again modestly hiding in its corner and we again pass. And this time we discover a couple of Roman ladies, we suppose they are Roman from their scanty clothing and the liberty the winds seem to be taking with it. The ladies have a sad and tearful look and evidently are not at present on good terms as they stand back to back against the opposite gate posts looking out of the corners of their eyes each in the direction of the other as if they would really like to make it all up and be good again. As we don't particularly enjoy tearful women even in pictures, we are about to pass on when we notice that the sign board over the gate-way reads "PROSTHODONTIA," and we conclude that the ladies are Greeks. As we turn the pages to the end of this series of articles we find a "tail-piece" in the form of an ear of corn in the husk. As it concludes one of Dr. Goslee's articles on Porcelain Crowns it must have significance. Guess what it is and you can have it. The next page is a real gem, it is one of those woodsy, pastoral, or mythical scenes that we are sure will fire the poetic impulses of the editor of the *Dental Review* as soon as he gets sight of it. There is a mocking bird in the tree and a beautiful female dressed in a kimono and an army overcoat playing a pair of Billy Sieberst's tin whistles to the evident surprise and disgust of a loosely robed and dirty-faced lass who has apparently lost her way and fallen asleep by the roadside. This picture is labeled ORTHODONTIA, but no one would ever suspect her we are confident. The next picture is labeled "Society Papers," and it represents Christopher Columbus reading one of his best sea serpent stories to an audience of six wigged and ruffled persons, only two of whom seem to be giving the slightest attention to what the reader is saying. If this picture illustrates society proceedings down east we are sorry for the men who spend midnight oil and much nervous energy in preparing enlightenment for them.

The next one is labeled "Society Discussions" and seems appropriate in design. Two learned looking gentlemen are evidently having a serious discussion about the occlusion of the teeth. There is an accompaniment of a considerable array of books, manuscripts, thirst quenchers, and a gavel. One of the gentlemen evidently has the better argument, and seems to be demonstrating from a skull which he holds in his hand that it is impossible to "jump the bite" while his antagonist with his hand over his own mouth is endeavoring to discredit the demonstration, or, judging by his frowning appearance, has either lost his argument or is holding onto himself until he can get a chance to upset the whole argument of his assailant. It is really a very appropriate design and does credit to its inventor. The "tail-piece" to this section is a flaming torch, which we suppose signifies that the discussion is not yet closed. As the prayer book says, "Good Lord Deliver Us."

As we proceed further we come to better art, at least to more classic designs. The editorial page is a gem, it is real Egyptian. It consists of a kind of portico of two columns supporting a lintal on which reclines and old lion—you can tell that he is an old lion by the way his tail is coiled up over his back—with a human face covered by an Egyptian foot-ball head gear. He seems to be contented, and should be as he is surrounded on all sides with palm leaves, bunches of grapes and other things calculated to appease the appetite and appeal to every sensuous taste. Just what it all symbolizes we can't quite make out, unless it represents the fulfillment of the dreams of good things in store for faithful hard-worked editors.

The "Editor's Corner" is also classic. Here the Editor is seated at a magnificent round table with a copy hook on one side and a cornucopia ink horn on the other. The office boy has evidently forgotten to return the waste paper basket as the papers lie scattered all over the floor. On the mantlepiece directly behind the editor sits a smart looking owl, a head of the sphinx and a whole lot of books. The Editor does not seem to have much use for the books, nor the owl either, but is earnestly engaged in pouring his words of wisdom into the ear of the blind and speechless Egyptian wonder. We are beginning to suspect that our readers may express the hope before they finish reading this that it might not be a bad idea for some other editors to have a sphinx conveniently near by.

The next heading is the "Book Reviews" which represents an old monk rapidly reading and passing on to the printer's devil the books in such haste that we fear his work can't be well done. The head piece "In Memorium" is a real work of art and should be done in oil, so the editor says, and he ought to know; it appears to be done on a palm branch with clasped hands and a bowed head. It is too sad to dwell longer on, and we trust the *Items* may not often have occasion to display it.

The series ends with a picture of a lugubrious individual with a shock of very black hair, dressed in a golf suit who seems to think he is in duty bound called upon to keep us posted as to the fact that the National Dental Association will meet July 30th, next, at Asheville, N. C. We trust other Journals may not feel slighted that we have devoted so much space to one particular journal; but when a new departure of so much worth comes under our notice we can't refrain from a suitable recognition. Our publishers have promised us a new dress of type this month, we wish they would add one or two pretty pictures, we should like a few.

